# Expression of tertiary lymphoid structure in deferred cytoreductive nephrectomy of metastatic renal cell carcinoma treated with nivolumab plus ipilimumab

**DOI:** 10.1002/iju5.12347

**Published:** 2021-08-16

**Authors:** Tomokazu Sazuka, Ayumi Fujimoto, Hiroaki Sato, Takayuki Arai, Yusuke Imamura, Shinichi Sakamoto, Jun‐ichiro Ikeda, Tomohiko Ichikawa

**Affiliations:** ^1^ Urology Graduate School of Medicine Chiba University Chiba Japan; ^2^ Diagnostic Pathology Graduate School of Medicine Chiba University Chiba Japan

**Keywords:** deferred cytoreductive nephrectomy, ipilimumab, nivolumab, renal cell carcinoma, tertiary lymphoid structure

## Abstract

**Introduction:**

Tertiary lymphoid structure expression and immune checkpoint inhibitors have been attracting attention, and their relationship with renal cell carcinoma is controversial.

**Case presentation:**

Two patients with nivolumab plus ipilimumab treatment response for metastatic renal cell carcinoma underwent cytoreductive nephrectomy and regional lymph node dissection. In both cases, the renal tumor site expressed tertiary lymphoid structures. Despite the absence of treatment after a deferred cytoreductive nephrectomy and the short postoperative observation period, the patients still survived.

**Conclusion:**

Tertiary lymphoid structures were expressed in deferred cytoreductive nephrectomy specimen in cases treated with nivolumab plus ipilimumab.

**Abbreviations & Acronyms**
ccRCC: clear cell renal cell carcinoma
DCN: deferred cytoreductive nephrectomy
ICI: immune checkpoint inhibitor
LND: lymph node dissection
mRCC: metastatic renal cell carcinoma
Nivo/Ipi: nivolumab plus ipilimumab
TLS: tertiary lymphoid structure
UCN: upfront cytoreductive nephrectomy

**Keynote message**

We report two patients with metastatic renal cell carcinoma who underwent deferred cytoreductive nephrectomy (DCN) and regional lymph node dissection after nivolumab plus ipilimumab treatment. They survived without any treatment after DCN, and tertiary lymphoid structures were expressed in the DCN specimens of both patients.

## Introduction

With the development of ICI, treatment outcomes for mRCC have sharply increased. In terms of the overall survival (hazard ratio; 95% confidence interval), Nivo/Ipi has remained superior to sunitinib in intention‐to‐treat (0.69; 0.59 to 0.81) and intermediate‐ and high‐risk patients (0.65; 0.54 to 0.78) after a minimum of 4 years of follow‐up. The rate of complete response in Nivo/Ipi is 10.7%.^1^ Recently, the effects of TLS expression and ICI have been attracting attention^2^ and remain controversial.

In this case report, we present two cases of mRCC who underwent DCN and regional LND. At the renal tumor site of both cases, TLS was expressed. Though they did not receive any treatment after DCN, they survived with no evidence of recurrence.

## Case presentation

### Case 1

An asymptomatic 76‐year‐old man presented with multiple lung metastases based on medical examinations. Dynamic computed tomography scan showed an 8 cm right renal mass, multiple lung metastases, bilateral adrenal metastases, and multiple pancreas metastases. Right renal tumor biopsy confirmed the existence of ccRCC, and immune cells including CD4, CD8, and CD20 were strongly expressed around the tumor (CD4: 5452, CD8: 3547, and CD20: 2123/mm^2^). PD‐L1‐positive cells were expressed in tumor cells, lymphocytes, and macrophages (PD‐L1: 20‐50 CPS; Figure S1). Hence, the patient was diagnosed with right mRCC. According to the IMDC risk classification, patient’s risk was poor (treatment from diagnosis, elevated neutrophil count, elevated platelet count). Thus, Nivo/Ipi was started without UCN.

**Fig. 1:**
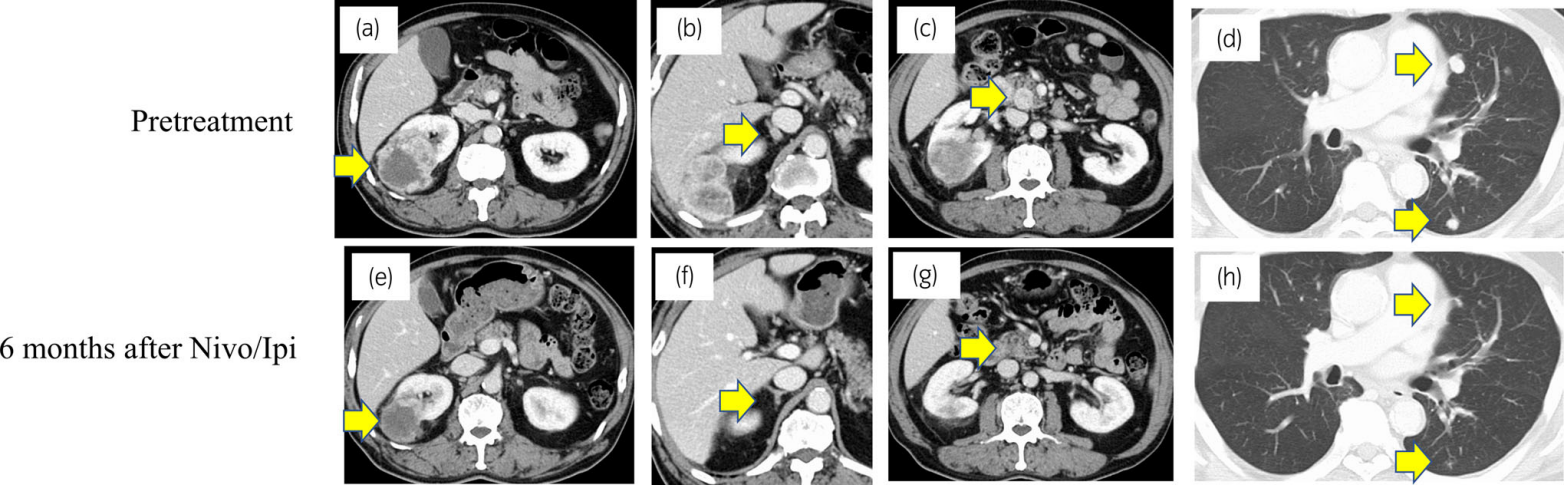
Computed tomography scan findings before treatment and 6 months after nivolumab plus ipilimumab treatment in primary site (a, e), right adrenal (b, f), pancreas (c, g), and lung metastases (d, h).

**Fig. 2:**
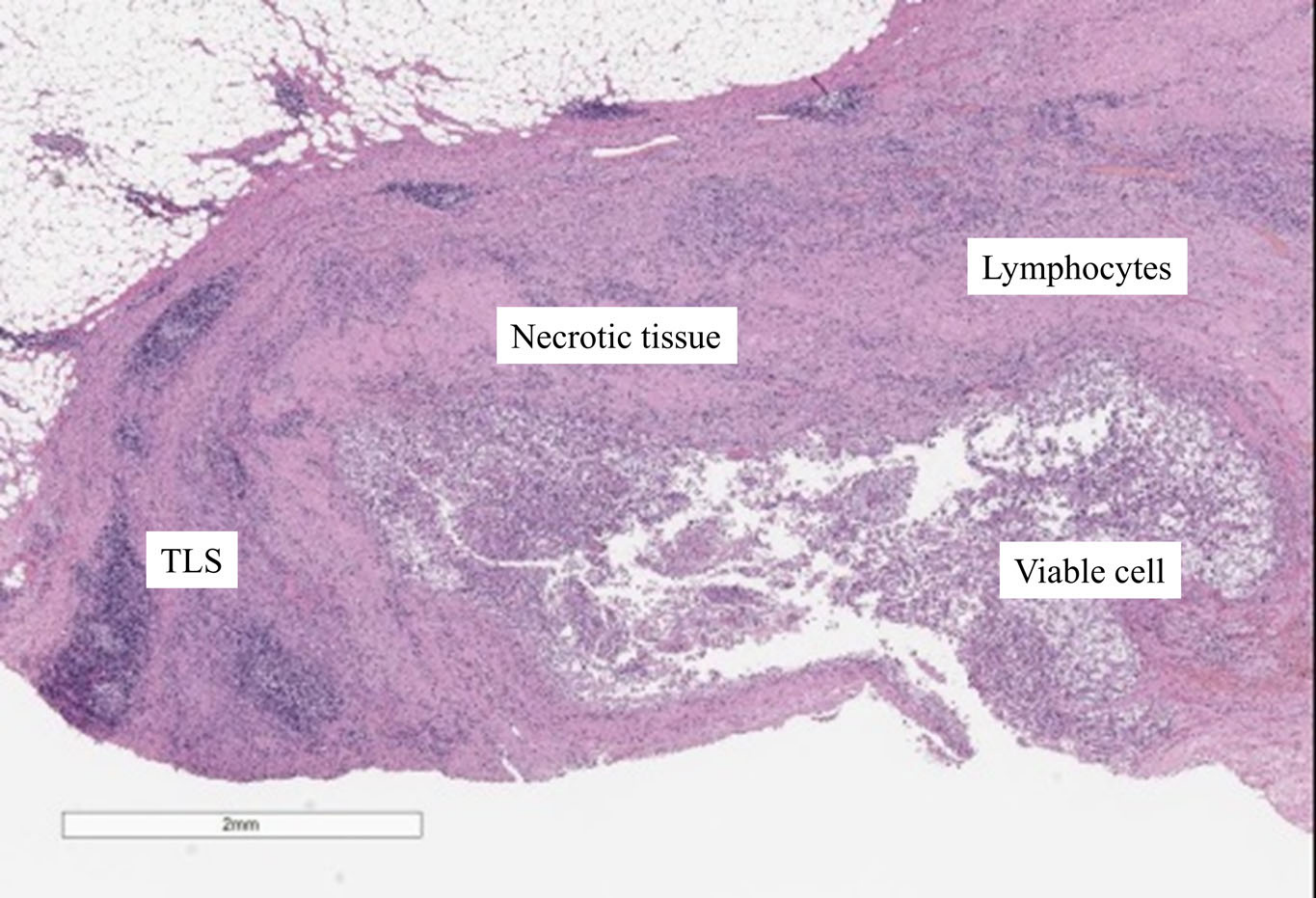
HE stains of the primary site at deferred cytoreductive nephrectomy in case 1. TLSs, viable cells, necrotic tissue, and lymphocytes are mentioned.

Eight months after Nivo/Ipi initiation, almost all metastatic sites disappeared (Fig. 1). Subsequently, we performed DCN, ipsilateral adrenalectomy, and regional LND by laparoscopic approach. The tumor strongly adhered to its surrounding. Few viable cells were present in the kidney and adrenal gland. Around the renal tumor, 10 TLSs were expressed. Immune cells including CD4^+^, CD8^+^, and CD20^+^ cells (CD4: 3997, CD8: 2419, and CD20: 614/mm^2^), and PD‐L1 (>50 CPS) were strongly expressed (Fig. 2, Figure S1). However, PD‐L1 was weakly expressed in the adrenal gland. No lymph node metastasis was found. After 15 postoperative months, no recurrence was observed.

### Case 2

A 60‐year‐old woman presented with neck pain and upper limb movement disorders. She underwent ossification of cervical posterior longitudinal ligament 5 years ago. At the orthopedics department, cervical spine biopsy and bone fixation were performed. Left kidney tumor biopsy revealed ccRCC. Immune cells including CD4, CD8, CD20, and PD‐L1‐positive cells were strongly expressed around the tumor (CD4: 6219, CD8: 5541, CD20: 2641/mm^2^, and PD‐L1: 20‐50 CPS; Figure S2) Hence, she was diagnosed with multiple bone metastases from left renal cell carcinoma. The IMDC risk was poor (treatment from diagnosis, low Karnofsky performance status, and elevated platelet count). Thus, external beam radiotherapy was performed on the cervical spine, and then Nivo/Ipi was started without UCN.

**Fig. 3:**
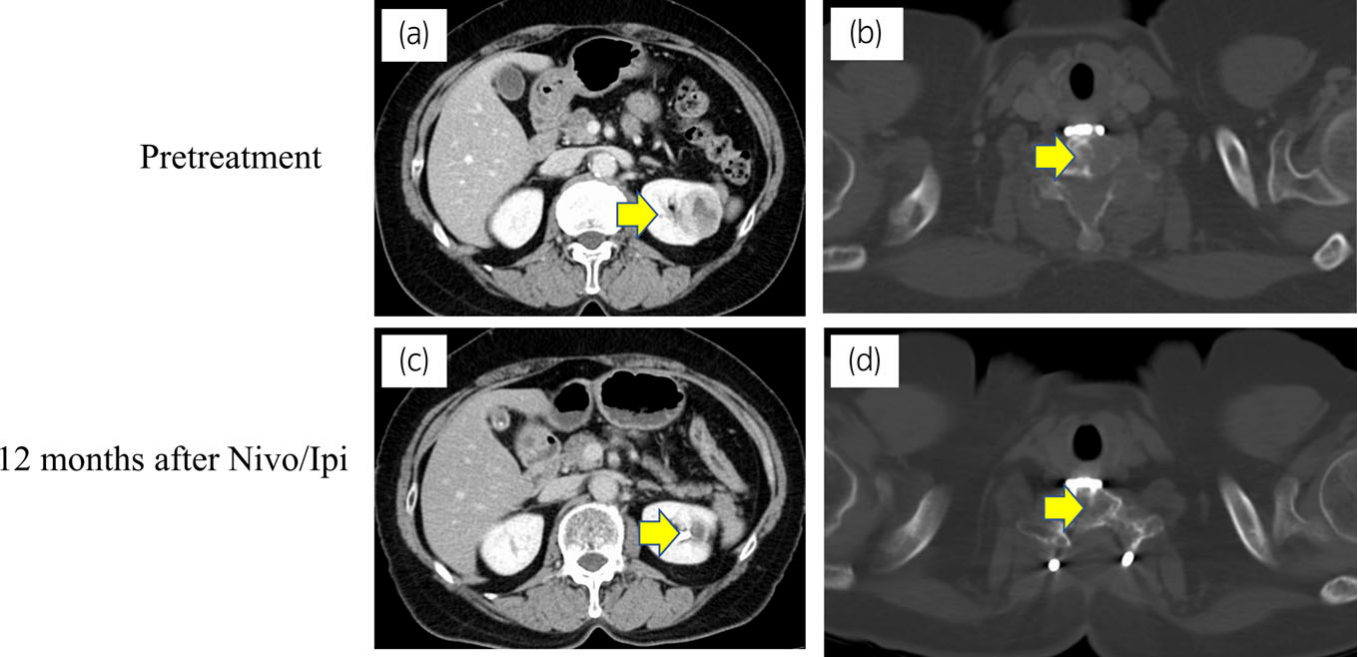
Computed tomography scan findings before treatment and 12 months after nivolumab plus ipilimumab treatment in the primary site (a, c) and cervical bone metastases (b, d).

**Fig. 4:**
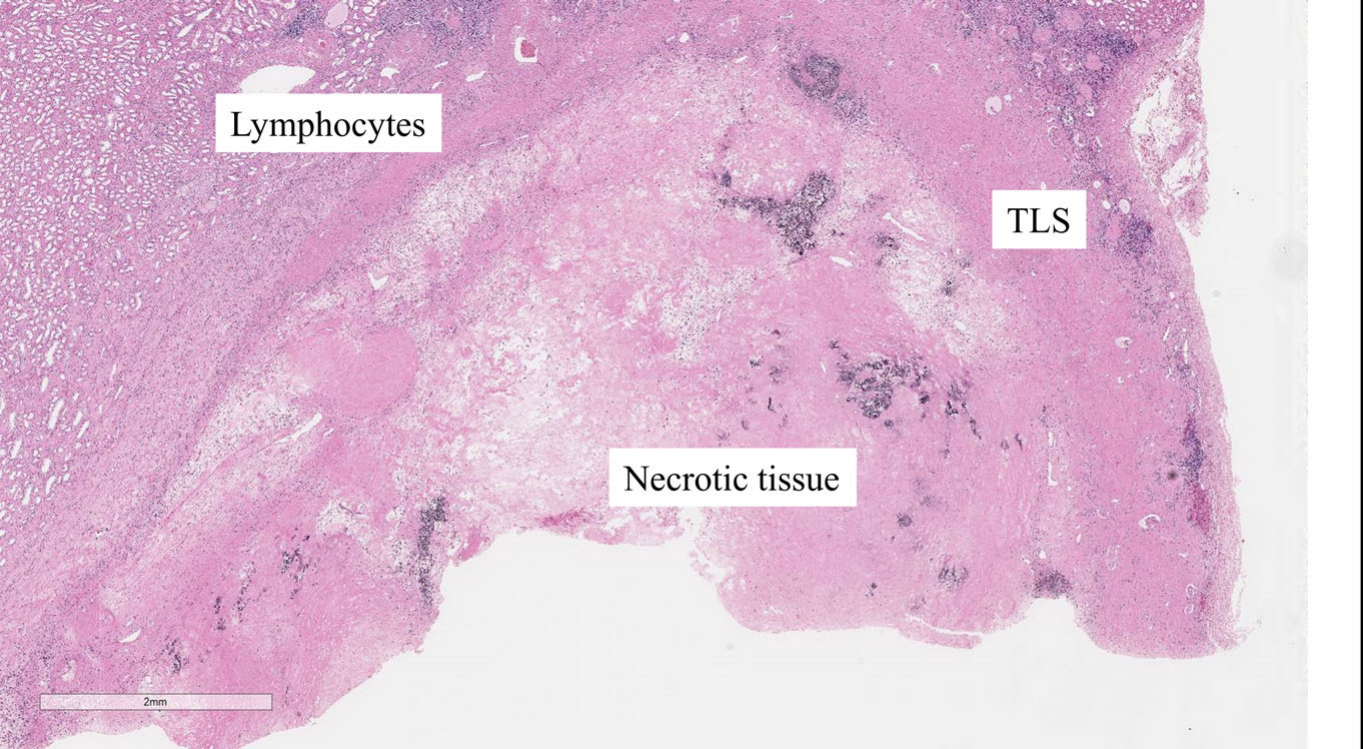
HE stains of the primary site at deferred cytoreductive nephrectomy in case 2. TLSs, necrotic tissue, and lymphocytes are mentioned.

Positron emission tomography with computed tomography confirmed no bone metastasis. Left RCC had almost disappeared (Fig. 3). After 1 year of Nivo/Ipi treatment, we performed DCN and regional LND by laparoscopic approach. The difficulty of surgery was the same as usual. The kidney showed no viable cells, but five TLSs were expressed around the necrotic tumor. Furthermore, immune cells including CD4^+^, CD8^+^, and CD20^+^ cells were strongly expressed (CD4: 4279, CD8: 4253, CD20: 1814/mm^2^) compared with PD‐L1 (<1 CPS; Fig. 4; Figure S2). No lymph node metastasis was found. After 6 postoperative months, no recurrence was observed.

## Discussion

In both cases, TLS was highly expressed at DCN. TLS is rarely expressed in patients at a low malignancy stage who underwent only nephrectomy or partial nephrectomy without immunotherapy. Various views on the expression of TLS and immunotherapy have been reported. The relationship between TLS and cancer immunotherapy remains unclear but has recently gained attention. TLS is ectopic lymphoid organ developed in nonlymphoid tissues at sites of chronic inflammation including tumors. The mechanisms that underlie the TLSs’ role in the adaptive antitumor immune response are being deciphered. Both T cells and B cells contribute to immunity against cancer.^2^ There were several TLSs near the necrotic tumor and a large number of lymphocytes in our two cases. In addition to lymphocyte infiltration from primary and secondary lymphoid structures, the presence of TLS near the tumor might have created a stronger immune attack.

Recently, Groeneveld *et␣al*. showed that as a surrogate for tumor TLS, CXCL13 expression could be a relevant predictive biomarker of response to ICI for advanced stage bladder cancer.^3^ In ccRCC, the abundance of CXCL13^+^CD8^+^ T cells was an independent prognosticator and a potential immunotherapeutic target marker.^4^ Considering the small materials used in the needle biopsy of our cases, the expression status of TLS before Nivo/Ipi remained unknown. However, in cases demonstrating therapeutic effects, large amounts of TLSs were expressed in the primary renal lesions. However, definition of TLS presence was not clear in this study. Further study is needed.

In both of our cases, lymphocyte infiltration was prominent in the primary lesion before Nivo/Ipi, indicating its potential to predict ICI response. In response cases, T‐cell and B‐cell lymphocytes may complementarily form TLS and attack tumor cells.

Currently, conclusions about regional LND in ICI treatment for mRCC remain inconclusive. LND performed during DCN after ICI treatment is also unreported. The lymph nodes are an important organ, wherein CTLA‐4 antibodies actively participate in ICI treatment. Regional lymph node is one of the most important parts, but its excision is predicted to inhibit the immune cycle. Though this study was observed for a short period postoperatively, no local or distant recurrences were noted, which suggests that LND performed at the same time as DCN may not cause recurrence in a short period of time. Thus, TLS may control cancers in the primary site and each metastatic site. However, this phenomenon still requires further studies. The meaning of DCN cannot be understood without long‐term observation. And there are no large‐scale studies examining the usefulness of DCN in the ICI era. A large, long‐term, prospective observational studies are going.^5^

## Conclusion

In our cases, immunohistochemistry, pretreatment renal lesion biopsy, and DCN lesion were performed. Although the postoperative observation period was short, patients survived without any treatment after DCN. Whether DCN contributed to long‐term survival or not, TLS was expressed in the DCN specimen of these cases treated with Nivo/Ipi.

## Conflict of interest

The authors declare no conflict of interest.

## Approval of the research protocol by an institutional reviewer board

The protocol for this research project has been approved by a suitably constituted Ethics Committee of the institution, and it conforms to the provisions of the Declaration of Helsinki.

## Informed consent

Informed consent for publication was obtained from the patients.

## Registry and the registration no. of the study/trial

This case report was approved by the Institutional Review Board of Chiba University Hospital (IRB No.2554).

## Supporting information


**Figure␣S1**. Immunohistochemical stains of the primary site at pretreatment, deferred cytoreductive nephrectomy, and tertiary lymphoid structure in case 1.

**Figure␣S2**. Immunohistochemical stains of the primary site at pretreatment, deferred cytoreductive nephrectomy, and tertiary lymphoid structure in case 2.
