# Supplementary figures and images for: Expression of tertiary lymphoid structure in deferred cytoreductive nephrectomy of metastatic renal cell carcinoma treated with nivolumab plus ipilimumab
Source: IJU Case Rep. 2021 Aug 16;4(6):355–8. doi: 10.1002/iju5.12347 (PMC8560443; doi:10.1002/iju5.12347)

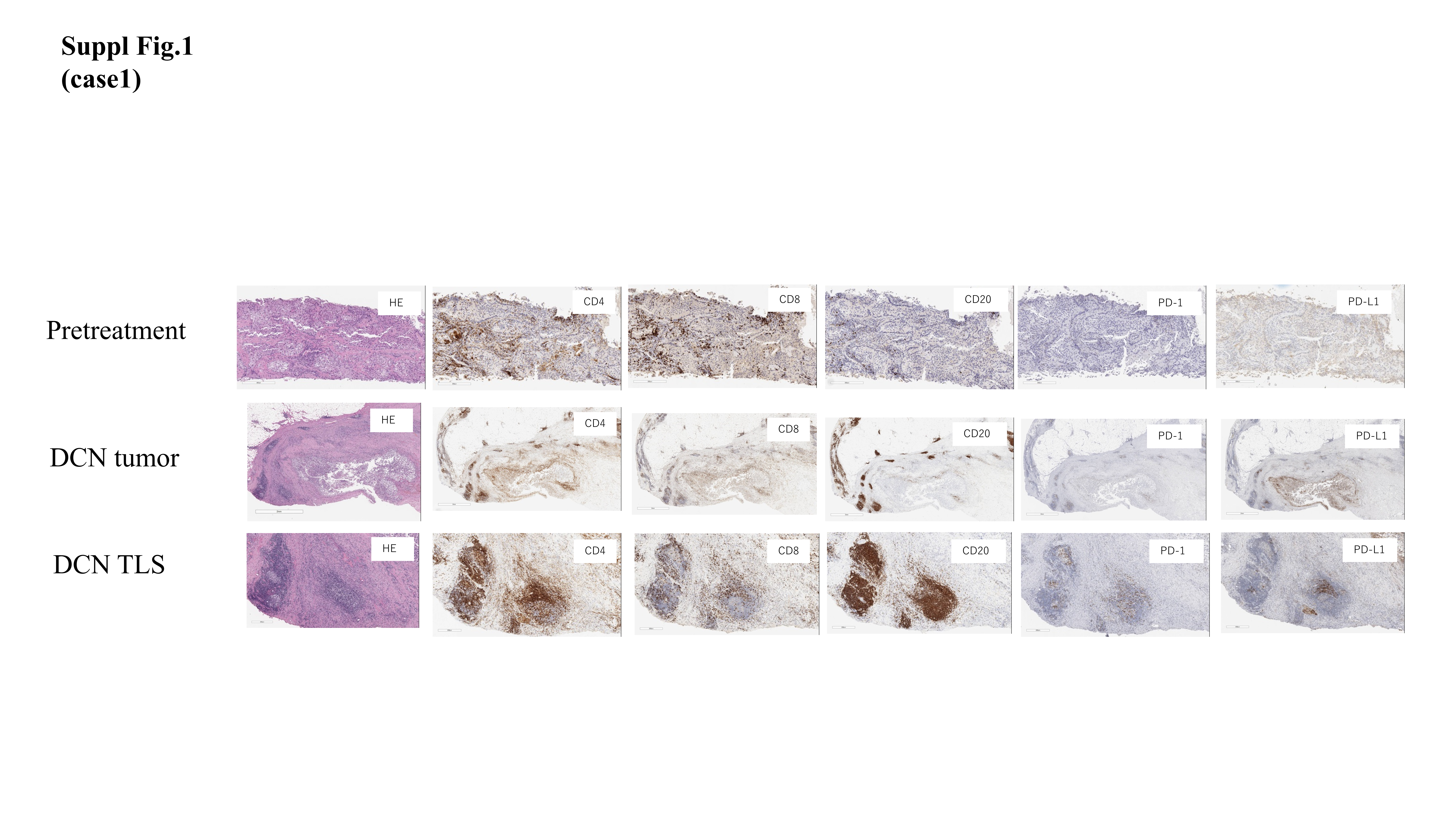

Supplement: Supplementary file 1 — Figure␣S1. Immunohistochemical stains of the primary site at pretreatment, deferred cytoreductive nephrectomy, and tertiary lymphoid structure in case 1. [file IJU5-4-355-s002.tif]

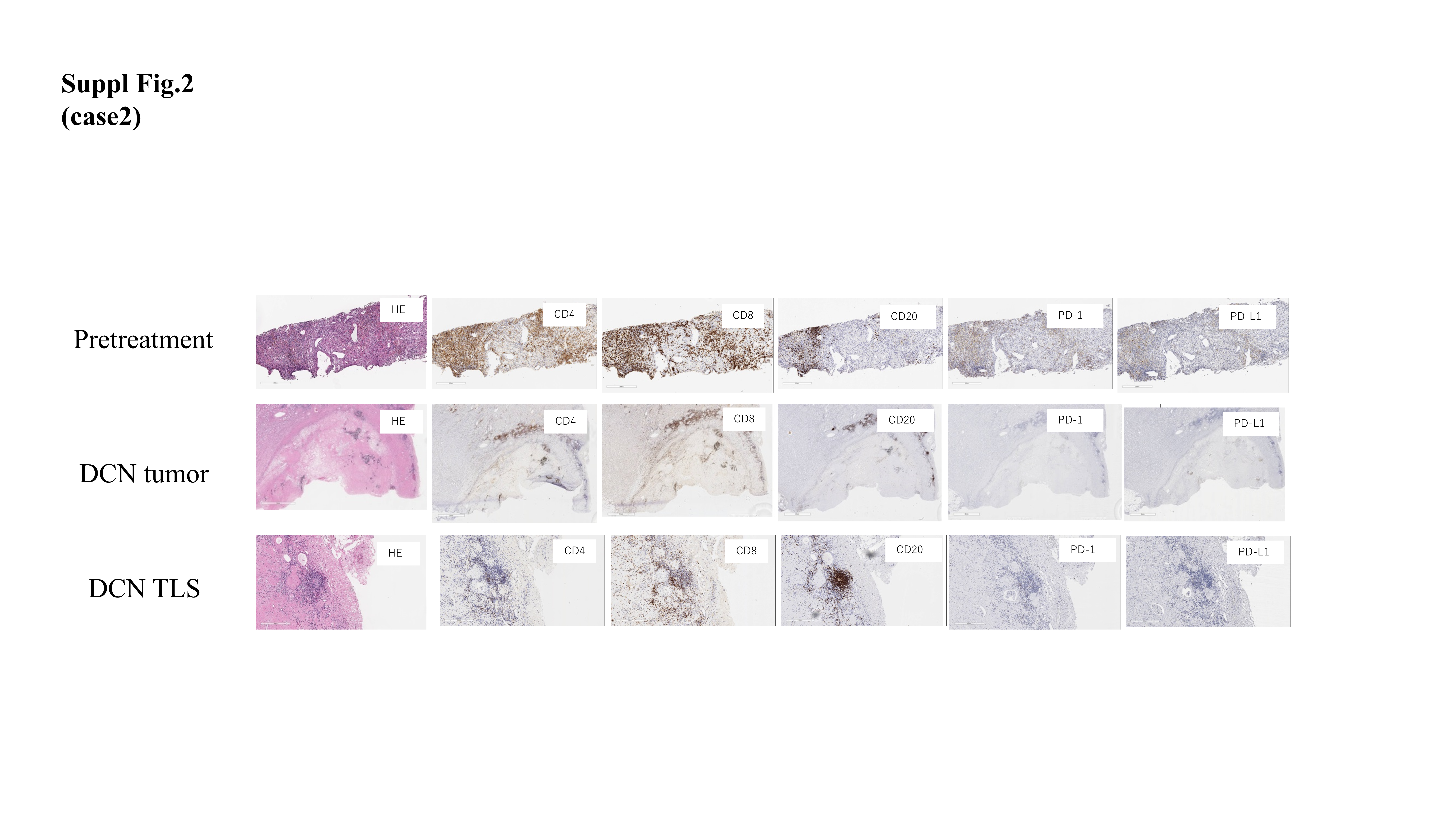

Supplement: Supplementary file 2 — Figure␣S2. Immunohistochemical stains of the primary site at pretreatment, deferred cytoreductive nephrectomy, and tertiary lymphoid structure in case 2. [file IJU5-4-355-s001.tif]
